# Modeling the Layer-by-Layer Growth of HKUST-1 Metal-Organic Framework Thin Films

**DOI:** 10.3390/nano11071631

**Published:** 2021-06-22

**Authors:** Qiang Zhang, Yohanes Pramudya, Wolfgang Wenzel, Christof Wöll

**Affiliations:** 1 Institute of Functional Interfaces, Karlsruhe Institute of Technology, 76344 Eggenstein-Leopoldshafen, Germany; qiang.zhang@kit.edu; 2 Institute of Nanotechnology, Karlsruhe Institute of Technology, 76344 Eggenstein-Leopoldshafen, Germany; yohanes.pramudya@kit.edu (Y.P.); wolfgang.wenzel@kit.edu (W.W.)

**Keywords:** HKUST-1, layer-by-layer, MD simulation

## Abstract

Metal organic frameworks have emerged as an important new class of materials with many applications, such as sensing, gas separation, drug delivery. In many cases, their performance is limited by structural defects, including vacancies and domain boundaries. In the case of MOF thin films, surface roughness can also have a pronounced influence on MOF-based device properties. Presently, there is little systematic knowledge about optimal growth conditions with regard to optimal morphologies for specific applications. In this work, we simulate the layer-by-layer (LbL) growth of the HKUST-1 MOF as a function of temperature and reactant concentration using a coarse-grained model that permits detailed insights into the growth mechanism. This model helps to understand the morphological features of HKUST-1 grown under different conditions and can be used to predict and optimize the temperature for the purpose of controlling the crystal quality and yield. It was found that reactant concentration affects the mass deposition rate, while its effect on the crystallinity of the generated HKUST-1 film is less pronounced. In addition, the effect of temperature on the surface roughness of the film can be divided into three regimes. Temperatures in the range from 10 to 129 °C allow better control of surface roughness and film thickness, while film growth in the range of 129 to 182 °C is characterized by a lower mass deposition rate per cycle and rougher surfaces. Finally, for T larger than 182 °C, the film grows slower, but in a smooth fashion. Furthermore, the potential effect of temperature on the crystallinity of LbL-grown HKUST-1 was quantified. To obtain high crystallinity, the operating temperature should preferably not exceed 57 °C, with an optimum around 28 °C, which agrees with experimental observations.

## 1. Introduction

Over the past decades, Metal-Organic Frameworks (MOF) have emerged at the forefront of solid-state chemistry due to their crystalline porous structure, versatile topology, and numerous potential applications [1,2]. MOFs are comprised of metal ions or clusters coordinated to di- or higher topic organic ligands to form solids with crystalline porous structures [3]. The standard MOF synthesis takes place using solvothermal conditions [4,5,6], where the crystallization of MOFs in solution is a spontaneous process where metal centers, nanoscale entities referred to as secondary building units (SBUs), bind to organic linkers. Alternatively, deposition on substrates can be achieved by using layer-by-layer techniques [7,8], which can generate thin films of high quality, with defect densities lower than the powdery products achieved by solvothermal synthesis [9]. By synthesizing MOFs from large libraries of metal clusters and organic ligands, researchers can create materials with a huge variety of different functionalities, such as gas separation [10,11], sensing [12,13,14], and drug delivery [15,16,17]. The structures of more than 70,000 MOFs have a largely empirical process of trial and error [18,19], and the resulting morphology of MOF particles and their structural quality are still difficult to control. Understanding the roles of synthetic parameters, such as temperature, pH, metal–ligand ratio, concentration, and solvents to optimize the operating conditions remains a crucial challenge for the controlled growth of MOFs.

Available theoretical strategies that can be considered to investigate the growth of MOFs include ab initio approaches, in particular, Density Functional Theory (DFT), force-field-based Molecular Dynamics (MD), Monte Carlo (MC) methods, as well as other numerical schemes such as the kinetic model [20], and the coupling of a kinetic model with a population balance model [21]. The crystallization of MOFs is a complex process that requires complex models. Unlike simple crystalline materials, where atoms can be modeled with little loss in accuracy as rigid spheres, the MOF building blocks have a more complex geometry and topology. Typically, a single unit cell of MOF contains hundreds or thousands of atoms making DFT calculations for the growth of such systems infeasible at present. Due to the complexity encountered in DFT calculations, classical force-fields are employed for the simulation of MOFs at the atomic level. Examples are the Universal force-field (UFF) [22], the MOF-FF [23], the UFF4MOF [24,25], force-fields derived from ab initio data, such as QuickFF [26], BTW-FF [27], etc. While these force-fields perform well in preserving the geometry of MOFs using predefined metal-ligand covalent bonds, they are not suitable for investigating the growth of MOFs when bond formation and breakage are involved. Reactive force fields, such as ReaxFF, that allow bond formation/breaking, unfortunately do not predict the proper geometry of most MOFs [28], and the computational performance of ReaxFF in most molecular dynamics programs (LAMMPS, GULP, etc.) is insufficient to reach the time-scales relevant for growth processes.

The investigation of MOF crystallization on the atomic level is computationally very expensive, and currently, only a few crystallization studies of MOFs with simple structures, like 2D planar (**sql**) topologies, or simple 3D topology, have been reported [29,30,31]. In addition, the definition and adjustment of force field parameters are problematic, especially when transition metal ions are involved. For the purpose of studying crystallization, many all-atom models, such as the non-bonded point charge model, generally struggle to reproduce the proper coordination geometry of MOFs [30]. Previous work by Yoneya et al. [29,32,33] employed the Cationic Dummy Atom (CDA) model to simulate the coordination-directed crystallization of 2D and 3D MOFs, as well as other spherical metal-organic complexes. The CDA model represents the metal ion implements with four pre-designed dummy atoms around the metal center in a square planar distribution to model metal coordination. This model was extended by Peter G. Kusalik et al. [30,31] to explore the crystallization process of an archetypal Zn-carboxylate system using an Enhanced Cationic Dummy Atom (ECDA) Zn-ion model. The ECDA model inherited the square planar distribution of the CDA model [34,35], while the charge distribution and force-field parameters of the ECDA model were adjusted. By performing simulations in a continuum solvent with Langevin dynamics (LD), the ECDA model was used to probe the crystallization of the Zn-carboxylate system in a longer time and length scale than those accessible by all-atom models. Nevertheless, due to the complexity of MOF crystallization, a MOF-2 structure with square topology could only be reproduced for very small systems. It takes approximately 200 ns for a system containing eight Zn-ions with four BTC and eight acetate ligands to obtain a nice square topology, while simulations over 1000 ns for a system containing 200 Zn-ions with 180 BTC ligands and 40 acetate ions fail to generate a globally organized structure, but achieve local organization.

Coarse-grained methods, in particular the Patchy Molecule (PM) model, therefore emerge as an effective option to investigate MOF growth. In these force fields, the coordination environment of MOF units is attained by the introduction of patchy sites predefined around a central particle. In order to reduce the number of calculations and to explore systems on larger temporal and spatial scales, coarse-grained MD (CGMD) schemes are a very promising approach. Jeetain Mittal and coworkers [36] used the PM model, which consists of Kern–Frenkel spheres fused together, to study the assembly of vertex-like building blocks with directional interactions. In their work, the planar crystallization of 2D covalent organic frameworks (COFs) was studied by simplifying the building blocks to hard spheres with attractive patches approximating the reactive ends of COFs. The directionality of covalent bond linkage was achieved by multiplying the attractive pair interactions with an orientation-dependent term. The authors used MC simulations of patchy building blocks to illustrate how the addition of chemical specificity via reacting functional sites can allow vertex-like building blocks to self-assemble into ordered crystallites structures. We have pursued this promising approach by developing a calibrated model specific for HKUST-1, a very popular, widely studied MOF with complex 3D geometry. We use the model study growth and nucleation in simulations modeling both one-pot and LbL synthesis to better understand MOF growth and to develop strategies for reducing defect densities and MOF thin-film roughness.

## 2. Model and Methods

In the following, we present a CGPM model for non-biased MD simulations to understand the growth of HKUST1 [4], a very popular prototype 3D MOF. The CGPM model was developed for the benzene-1,3,5-tricarboxylate linkers and Cu-paddlewheel SBUs, the constituents of this MOF. Both building blocks were treated as rigid bodies, with no parameters for internal bonds, angles and torsion potentials. All simulations in this work were performed using HooMD-2.5.2 [37,38], and the building blocks of coarse-grained (CG) structures of HKUST-1 were prepared with AuToGraFS [39].

### 2.1. Patchy Models

**Figure 1:**
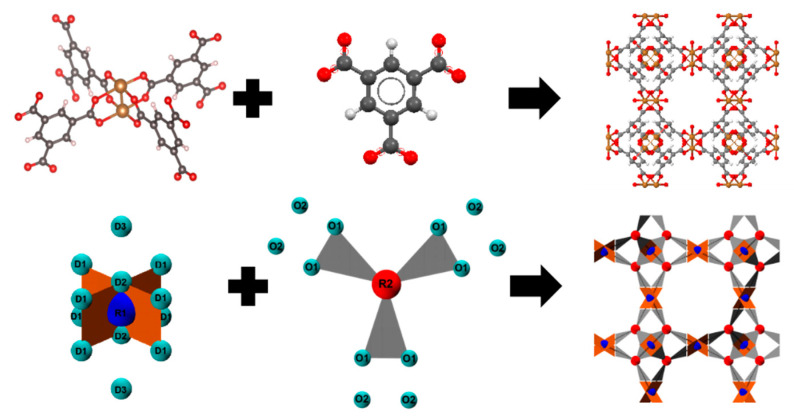
AAMD (**top**) and CGPM (**bottom**) models for precursors and HKUST-1. CGPM SBU color scheme: Blue: R1, Cyan: D1, D2 and D3; CGPM Ligand color scheme: Red: R2, Cyan: O1 and O2. The CGPM model of SBU represents 2 Cu ions.

The HKUST-1 framework is formed by connecting two types of building blocks; see Figure 1. The first building block is shaped like a paddlewheel (PW), while the second building block represents a benzene-1,3,5-tricarboxylate (BTC) ligand. The PW unit consists of two Cu^2+^ atoms bonded to the oxygens of the BTC ligands, as illustrated in Figure 1. For the CGMD simulations used here to study the crystallization of HKUST-1, the two building blocks were treated as rigid particles with patchy sites. In the context of the CGPM model, the aggregation of building blocks to form HKUST-1 particles occurs by attaching a patchy site of the SBU to the patchy site of BTC; see Figure 1.

To simulate the dynamics on the individual building blocks, the mass of each building block is concentrated on its center of mass; R1 in case of SBU, and R2 for BTC (see Figure 1). Forces arising from interactions with other SBUs cause torques, which are determined assuming the building blocks to be completely rigid. Most experimental synthesis procedures yield MOF material containing water molecules coordinated to the axial positions (D2 in Figure 1) of the Cu^2+^ ions in the PW units. According to experimental thermal gravimetric analysis (TGA), these weakly bound species are released from the framework upon heating the HKUST-1 from 25 to 120 °C in an inert atmosphere. All simulations carried out in this work refer to the activated case, i.e., the SBU mass amounts to 2 × 63.5 amu (Cu). The BTC mass equals 207 amu. Five types of patchy sites, namely D1, D2, D3, O1, and O2, are attached around the rigid centers to approximate the coordination environment, as shown in Figure 1.

In a perfect HKUST-1 structure, 4 BTC ligands are attached to a PW SBU by forming two (O1-D1) and two (O2-D2) pairs, i.e., O1 is placed at a D1 position and O2 at a D2 position. These patchy sites reflect the directionality of the coordination bond between the carboxylate groups and the Cu^2+^ ions. The patchy D3 position introduces a repulsive interaction to avoid unwanted bonding from top and bottom of the SBU, as demonstrated in Figure S2. When the D3 patchy sites were omitted, the solids obtained by our simulations did not have the HKUST-1 or any other crystalline structure but were amorphous.

**Figure 2:**
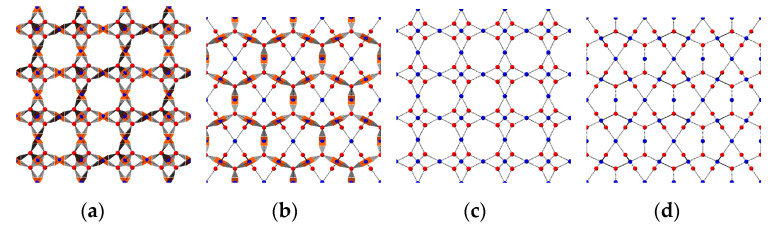
(**a**) Structure of HKUST-1 in the CGPM model in the (100) crystal plane; (**b**) HKUST-1 using CGPM model in the (110) plane; (**c**) simplified tbo net of HKUST-1 in the (100) plane; (**d**) simplified tbo net of HKUST-1 in the (110) plane.

The interactions of patchy sites can be classified into three types depending on the form of interactions: attractive pairs, non-interactive pairs, and repulsive pairs. The attractive pairs interact only through attractive forces; the paired patchy sites, which pair O1-D1 and O2-D2, are allowed to overlap. Some pairs, such as O2-O2, which belong to different rigid bodies, do not interact (see Figure S1). The repulsive patchy pairs are set up to prevent overlap among rigid bodies and are applied to all remaining patchy pairs. In Figure 2, we illustrate the **tbo** topology [40] of HKUST-1 along the (100) and (110) crystallographic directions to demonstrate how the crystal structure is represented by the model.

### 2.2. Force Field

The reactive patchy pairs interact via a Truncated Morse potential (TMORSE), while repulsive patchy pairs interact via a Weeks-Chandler-Andersen (WCA) [41] potential. The TMORSE potential adopted in our simulation is defined by:

(1) $$\begin{array}{c} \alpha =\sqrt{k_{e}/2D_{e}}\\ \;U_{TMORSE}=\{\begin{array}{c} D_{e}\cdot {\left(1-e^{-\alpha \cdot r}\right)}^{2}-D_{e},\;\;\;\;\;if\;r\;\leq r_{cut}\\ 0,\;\;\;\;\;\;\;\;\;\;\;\;\;\;\;\;\;\;\;\;\;\;\;\;\;\;\;\;\;\;\;\;\;\;\;\;\;\;\;\;\;\;\;\;\;otherwise \end{array} \end{array}$$

Here, *r* is the distance between CG particles, *r_cut_* is the cutoff distance, *D_e_* is the dissociation energy, *k_e_* is the harmonic force constant at the minimum of the well (equal to the harmonic force constant of coordination bond), and *α* is a parameter controlling the width of the potential curve. TMORSE is a popular interaction potential yielding realistic vibrational motions of molecules and a good description of bond formation and bond-breaking processes [42].

The interaction between repulsive patchy pairs is described by a WCA potential, which is defined by:

(2) $$U_{WCA}=\{\begin{array}{c} 4\varepsilon_{WCA}\left[{\left(\frac{\sigma }{r}\right)}^{12}-{\left(\frac{\sigma }{r}\right)}^{6}\right]+\varepsilon_{WCA},\;\;\;\;\;if\;r\;\leq 2^{1/6}\sigma \\ 0,\;\;\;\;\;\;\;\;\;\;\;\;\;\;\;\;\;\;\;\;\;\;\;\;\;\;\;\;\;\;\;\;\;\;\;\;\;\;\;\;\;\;\;\;\;\;\;\;\;\;\;\;\;\;\;\;\;\;\;\;otherwise \end{array}$$

The WCA potential is a Lennard-Jones (LJ) potential, shifted upward by *ε_WCA_*, and set to zero when the pair distance *r* is greater than 2^1/6^*σ*. In our simulation, *r_cut_* equals 2^1/6^*σ*; it was determined from the radial distribution function (RDF) of coarse-grained HKUST-1, and σ was determined by *r_cut_*/2^1/6^. By adjusting the *ε_WCA_*, the repulsive strength can be adjusted to avoid overlap of the units in the simulations.

We note that the depth of the potential well ε between reactive patchy pairs can be used to set the energy scale of the model, i.e., to calibrate the temperature. Simulations with *ε* = 1 are referred to as simulations in reduced temperature units. The depth of potential well between patchy pairs O1-D1 and O2-D2 were both set to value of 50 times the energy unit *ε*. The cutoff distance set between reactive patchy pairs has to be chosen with care in order to prevent unwanted interactions with the patchy sites that are located further away than the nearest neighbors, but it does not affect the energetics of the system strongly. Langevin dynamics was adopted in all simulations to mimic the viscous aspect of the solvent; the system temperature was maintained with the Langevin thermostat. The simulation time step d*t* was 0.002 *τ*, the time unit in HOOMD simulations.

### 2.3. Crystallinity Quantification Algorithm

The radial distribution function, RDF or g(r), is defined by:

(3) $$g\left(r\right)=\frac{dn_{r}}{\begin{array}{c} 4\pi r^{2}dr\rho \end{array}}$$

The crystallinity parameter in this work was calculated by *n_all_/n_crystal_*, where *n_all_* is the number of particles within a shell spanning distance *r*_1_ to *r*_2_, and *r_crystal_* is the number of particles at crystalline cites within a shell spanning from distance *r*_1_ to *r*_2_, as shown in Equation (4):

(4) $$\begin{array}{c} n_{all}=\underset{r\in all}{\sum }4\pi r^{2}dr\rho \cdot g\left(r\right)\\ n_{\mathrm{crystal}}=\underset{r\in crystal}{\sum }4\pi r^{2}dr\rho \cdot g\left(r\right)\\ Crystallinity=\frac{n_{all}}{n_{crystal}}*100\%=\frac{\sum_{r\in all}4\pi r^{2}dr\rho \cdot g\left(r\right)}{\sum_{r\in crystal}4\pi r^{2}dr\rho \cdot g\left(r\right)}*100\% \end{array}$$

The minimum and maximum distances *r* were chosen carefully to include the secondary peaks only during RDF analysis, since peak width and intensity of secondary peaks reveal the long-range ordering. The crystallinity in our work was calculated with respect to pairs R1-R1, R1-R2 and R2-R2 since they represent the respective building units.

## 3. Results and Discussion

### 3.1. Thermal Stability Analysis

In our model, the unit of energy in simulation is *ε*, which we determine by fitting to the experimental data for the HKUST-1 melting point. The available experimental data from thermal gravimetric analysis (TGA) and high-temperature single-crystal diffractometry indicate that HKUST-1 is stable up to 240 °C [4]. In the simulations, the thermal stability was determined by monitoring the change in the number of coordination bonds as a function of temperature.

**Figure 3:**
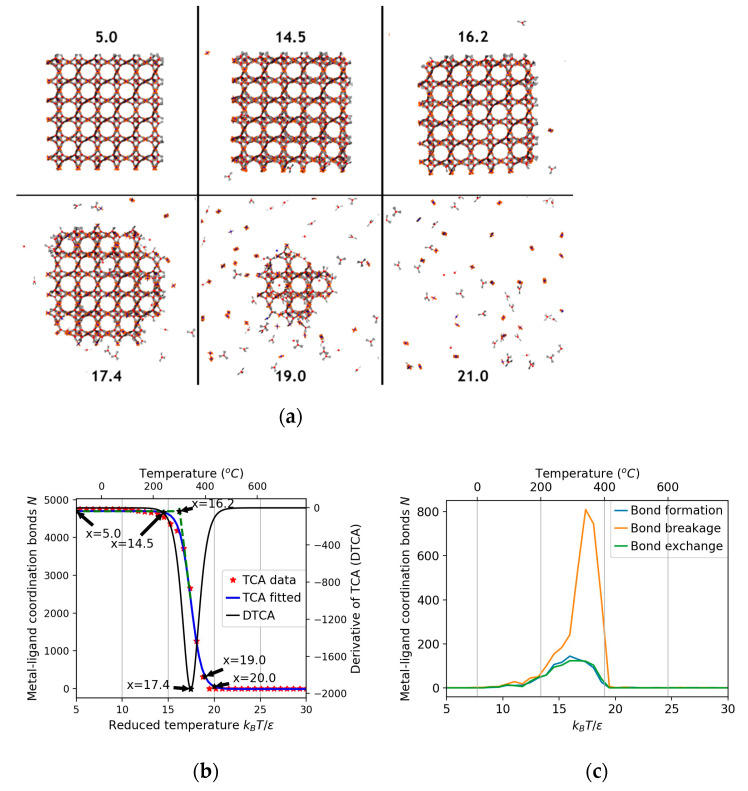
(**a**) The changes of HKUST-1 structure as T* increases from 5 to 30 in reduced unit; x-values indicate the reduced simulation temperatures. (**b**) Number of coordination bonds in the system as a function of the reduced temperature; 14.5 is the onset temperature which measures the thermal stability, 17.4 is the temperature which corresponds to the maximum decomposition rate. (**c**) The number of bonds formed, broken, and exchanged at different T*.

Simulations were performed with a 3 × 3 × 3 crystal HKUST-1 placed in the center of a (50 nm)^3^ simulation box, and the temperature in reduced units was increased in a continuous fashion from 5 to 30. Representative structures from the simulations are summarized in Figure 3a. We perform two types of analysis for the thermal stability: Figure 3b shows a rapid crossover from a high coordination number to almost no coordination as the temperature is increased. Figure 3c shows the number of bonds formed, broken and exchanged at different T*. In analogy to TGA analysis, we fitted the middle point of the crossover region to determine the transition temperature T* as approximately 14.5; for higher temperatures, the initially cubic HKUST-1 crystal evolved into a spherical shape and started to decompose rapidly until it was completely dissolved. From a comparison to the experimentally observed values for the onset of decomposition, a value for the energy unit *ε* of 0.294 kJ mol^−1^ was determined.

### 3.2. 2D Growth of HKUST-1 on Planar Substrates

LbL synthesis is widely used for the fabrication of MOF thin films, and a particular target of the present work was to consider how the parameters of this step-wise deposition process influence thin-film morphology, defect density, and surface roughness in the case of HKUST-1. Probably the most important ingredient of the LbL method is that reactants are not mixed as for solvothermal synthesis, but are kept apart. The growth of HKUST-1 thin film is carried out by subsequent immersion of appropriately functionalized substrates into solutions of Cu acetate and benzene-1,3,5-tricarboxylate acid. The individual immersion steps are separated by rinsing with the solvent (ethanol). We have carried out simulations where the immersion in the SBU or BTC containing solutions was mimicked having temporarily either only free SBU or BTC in the simulation. In the following two sections we will first discuss the effect of concentration on the resulting crystallinity and the resulting mass deposition rate per cycle. Subsequently, we will discuss the effect of temperature by examining the surface roughness, the mass deposition rate per cycle, and crystallinity.

In each addition cycle, the production time is equal to the simulation time in LbL simulations. There is no requirement of calibration or thermalization time since the precursors cannot move or deform as a result of using the rigid-body model. The production time in each cycle was 11 ns for the formation of the SBU and 22 ns for the formation of the bonding of the BTC linker to the SBU.

#### 3.2.1. The Effect of Reactant Concentration

In the following, we investigate the effect of reactant concentration on the growth process. We note that concentration in planar LbL growth refers to the local concentration in solution near the surface rather than the overall concentrations. In the growth process on the 2D surface, precursors in a thin layer just above the surface must be supplemented from the bulk phase due to the concentration gradient caused by the depletion of precursors during growth. When the number of precursors is constant throughout the MD simulation, free precursors integrated into the crystal reduce the concentration in solution and cannot be replaced from a bulk phase. We overcome this problem in the simulations by adding a specified number of precursors initially. We monitor the mass deposition rate per cycle and the morphology of the final structure produced under four different concentrations.

Slab boundary conditions were adopted in planar growth simulations, with one layer of BTC (-COOH terminated) frozen on top of the bottom wall for precursor growth. Between each addition step, the free SBUs, BTCs, and small clusters not adhering to the substrate were removed to mimic the rinsing operation in the experiment. The size of the simulation box is 5.29 × 5.29 × 10.00 nm, such that the area perpendicular to the substrate accommodates 2 × 2 HKUST-1 unit cells. All simulations were performed in an NVT ensemble with a time step of 0.002 *τ* and the temperature set to 25 °C. The number of initial precursors (SBU or BTC) added per cycle amounted to 32, 48, 64, and 80. Note that even the minimum number of precursors added should be sufficient to cover all the sites where the surface can grow. A full cycle consisted of exposure to SBU and subsequent exposure to BTC, and three cycles were studied for each parameter set. Each individual simulation in this protocol was continued until the change in total energy per unit time was less than 2%.

**Figure 4:**
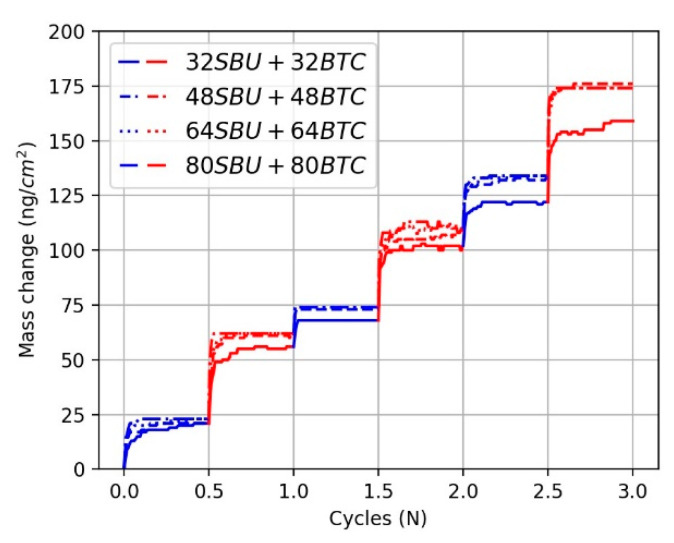
LbL mass increase during 3 growth cycles, in which blue indicates the SBU addition step and red indicates the BTC addition step. The number of precursors added each cycle is indicated in the inset. When the number of precursors added is greater than or equal to 48 each, there is no significant change in the mass increase.

**Figure 5:**
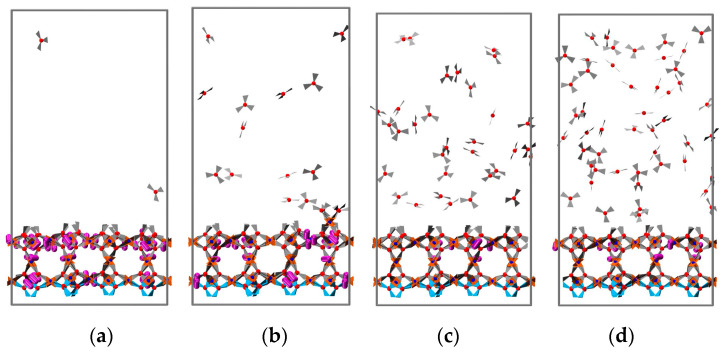
Snapshots for structures obtained for the four different concentrations with the seed layer shown in blue. (**a**) When 32 precursors were added per cycle; (**b**) 48 precursors added per cycle; (**c**) 64 precursors added per cycle; (**d**) 80 precursors added per cycle.

The mass deposition rates per cycle for the three additional cycles are summarized in Figure 4. The resulting mass deposition rate increases when the number of initially added precursors increases from 32 to 48, while the mass deposition rates per cycle are quite similar when the number of added precursors is 48 precursors or more. Hence, more than 48 precursors are needed each cycle in order to fully cover a 2 × 2 unit of HKUST-1 at around 25 °C. The resulting structures are illustrated in Figure 5, in which the purple cylinders represent the vacant sites in the grown HKUST-1 film.

Experimental work by Schäfer et al. [20] focusing on HKUST-I growth from Cu-nitrate and BTC under homogeneous synthesis conditions reported that the increase in Cu^2+^ concentration resulted in a greater CuBTC growth rate (as observed here), while an increase in BTC concentration lead to lower CuBTC growth rates. The latter finding is at variance with our observations, but it has to be noted that our simulations are carried out using LbL growth conditions (i.e., reactants are kept separate). In addition, the metal sources are different. Previous work has shown that LbL growth depends strongly on the metal source [43,44].

The effect on the crystallinity of the resulting HKUST-1 film was investigated by computing the RDF, which is summarized in Figure S3. It was found that the peak width and intensity of RDF distributions under different concentrations are similar; therefore, the effect of concentration on the crystallinity appears to be relatively weak.

#### 3.2.2. The Effect of Temperature

**Figure 6:**
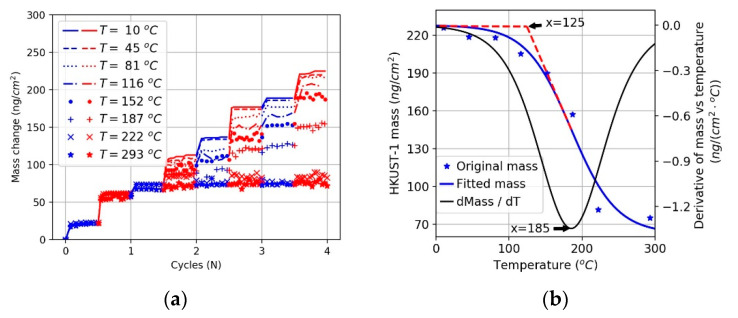
(**a**) Mass change after 4 addition cycles under 4 different temperature conditions. When temperature chosen falls within 10–81 °C, the accumulated mass deposition after 4 cycles has no significant difference, hile a further increase of temperature could result in gradual decrease of mass deposition. (**b**) The accumulated mass deposition after 4 cycles under different temperatures, the mass deposition rate starting to decrease at 125 °C.

When investigating the effect of temperature, the concentration was set to 64 precursors (SBUs or BTCs) per cycle. The potential effect of temperature on the mass deposition, morphology, and crystallinity of the resulting structure was investigated in eight cases for temperatures of 10, 45, 81, 116, 152, 187, and 222 to 293 °C. For each temperature condition, we simulated LbL growth as above for four cycles. The mass deposition as a function of SBU or BTC addition cycles is summarized in Figure 6a, where the blue sections represent the SBU addition cycles and the red sections the BTC addition cycles, and results obtained at different temperatures are distinguished by different line styles.

The amounts of deposited mass per cycle per unit area were very similar at temperatures ranging from 10 to 81 °C, showing only a small difference in cumulative mass deposition after four cycles. For temperatures above 116 °C, a substantial decrease in the accumulated deposited mass after four cycles was observed. For temperatures larger than 152 °C, it becomes more difficult for building units to attach to the surface, which leads to a further decrease in the HKUST-1 mass deposition rate. However, the mass increase during the first cycle shows only a very weak dependence on temperature. Noteworthy, this result is in perfect agreement with the experimental finding reported by Mark Allendorf and coworkers [45], in that the initial Cu^2+^ deposition step has a rate that is independent of Cu(OAc)_2_ concentration and temperature. The accumulated mass deposition after four cycles has been summarized in Figure 6b, in which we indicated an extrapolated onset temperature of 125 °C as the highest operating temperature that yields a substantial amount of HKUST-1 material.

**Figure 7:**
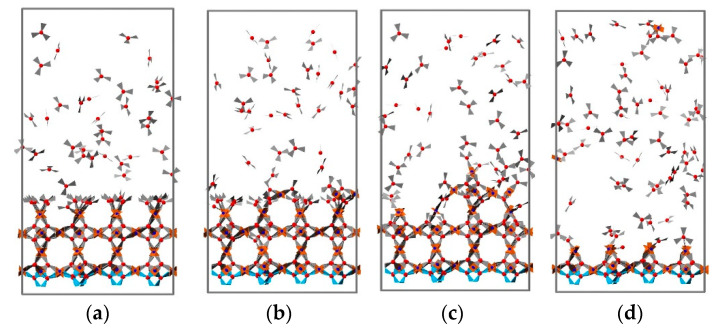
Snapshot taken for structure obtained at different temperatures: (**a**) 10 °C; (**b**) 81 °C; (**c**) 152 °C; (**d**) 222 °C. The frozen BTC seed layer is drawn in blue.

**Figure 8:**
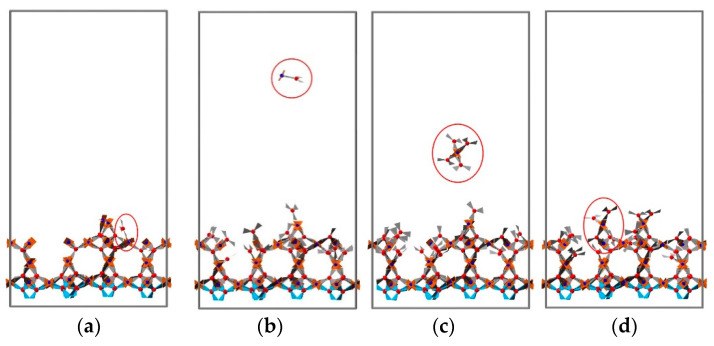
(**a**–**d**) Snapshots taken from the trajectory of the third cycles at 152 °C. The loosely attached building block breaks away from the surface; it then grows in bulk and rearranges itself to the surface in small clusters rather than free precursors. This process contributes to the inhomogeneous growth of HKUST-1 film. The rest of the free building units are omitted for clarity, and the frozen BTC layer is drawn in blue.

**Figure 9:**
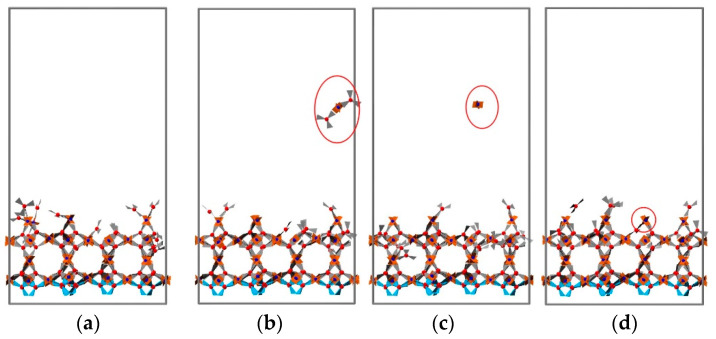
(**a**–**d**) Snapshots taken from the trajectory of fourth addition cycles at 187 °C; the loosely attached building block breaks away from the surface, the cluster then decomposes in bulk, and its components reattach to the surface, just as free precursors. This process heals the surface and results in smaller surface roughness. The rest of the free building units are omitted for clarity, and the frozen BTC layer is drawn in blue.

**Figure 10:**
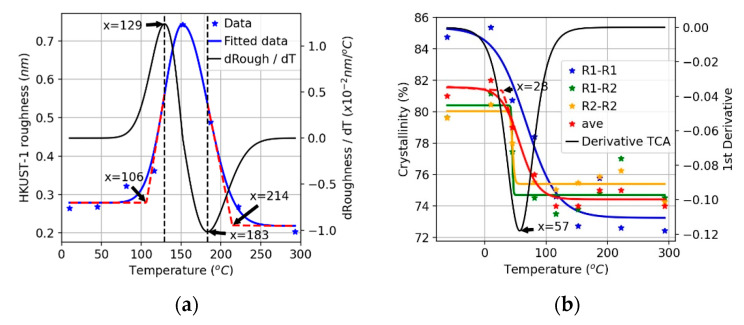
(**a**) Roughness calculated under different temperature; (**b**) Crystallinity calculated based on RDF distributions of pair R1-R1, R1-R2, and R2-R2.

For practical applications, the roughness of MOF thin films deposited using the LbL method is a very important parameter, especially for advanced applications of MOFs where, e.g., sharp heterojunctions are required [46] or very flat surfaces are needed. In order to investigate the effect of deposition temperature, simulations of four deposition cycles were carried out, and the final structures were analyzed for eight different temperatures. Four typical snapshots recorded for temperatures of 10, 81, 152, and 222 °C are displayed in Figure 7. Analysis of the results permits the identification of three different growth regimes. At temperatures less than 129 °C, the surface roughness is small, as evident from snapshots (a) and (b). For temperatures between 129 and 183 °C, the growth rate is smaller and the roughness larger. We explain this finding by a higher probability of bond breakage at these temperatures, where loosely attached building units are more likely to be pulled off the surface by their counterpart units added in the next addition cycle. The removed parts continue to grow in bulk and then rearrange onto the surface as small clusters rather than as free precursors. This contributes to the inhomogeneous growth of the HKUST-1 film, as illustrated in Figure 8. When the temperature is greater than 183 °C, both the growth rate and the roughness are reduced. In this regime, the bond formation probability decreases further, and bond breakage probability becomes more pronounced. When loosely attached building units break away from the surface, they are more likely to decompose further. Precursors tend to eventually rearrange and heal on the surface as monomers; this process is demonstrated in Figure 9. The variation of surface roughness as a function of temperature and the three regimes are summarized in Figure 10a.

In fact, the increased roughness of the deposited layer at increased temperatures observed in our calculations could be related to the cluster formation process proposed in the previous experimental work by Schäfer et al. [20]. Note, however, that in this experimental work, solvothermal synthesis conditions were used, while here, we focus on LbL growth.

Using the RDF in Figure S4, the crystallinity for each temperature was calculated and fitted; the results are summarized in Figure 10. Even considering such a large temperature range, the difference in the calculated crystallinity is not significant (less than 10%). This finding deviates substantially from observations reported for MOF nucleation in the context of solvothermal synthesis. From the point of view of obtaining HKUST-1 film of higher crystallinity, the preferred operating temperature is less than 57 °C, and optimally less than 28 °C, including the room temperature, which is quite often used in the experiment.

## 4. Conclusions

In our study, a simulation method was developed to describe the layer-by-layer deposition of MOF thin films on solid substrates. Using a specifically developed CGPM model for HKUST-1 together with a non-biased MD simulation method, we successfully simulated the growth of HKUST-, reproducing earlier experimental findings [45]. Our model helps to understand the morphological features of HKUST-1 thin films grown under different concentration and temperature conditions and can be used to predict and optimize the temperature for the purpose of controlling growth quality and deposition yield. For the study of planar LbL growth, we observed a strong correlation between concentration and the mass deposition rate per cycle up to a concentration where saturation is observed. We find that the concentration of the precursors has a limited effect on the crystallinity of the resulting planar HKUST-1. The effect of temperature on LbL growth of HKUST-1 was investigated by simulating eight different temperature conditions from 10 to 293 °C. We were able to identify three different growth regimes with different surface roughness and film thickness when grown on a perfect and flat SAM substrate. Regime 1, in the range of 10 to 129 °C, allows good control of surface roughness and film thickness. In regime 2, in the range of 129–183 °C, we observed a smaller mass deposition and rougher surfaces. This regime is not conducive to controlling the surface roughness. In regime 3, for temperatures higher than 183 °C, both the surface roughness and film deposition rate are reduced simultaneously. In order to obtain large mass deposition and good crystal quality, the operating temperature should range from room temperature to 57 °C, which is in good agreement with the experimental LbL temperature conditions. On the basis of our simulations, we were able to identify the role of small clusters in the surface as detrimental to crystal growth in the intermediate temperature range. Such clusters are unlikely to nucleate spontaneously in solution but can form on and break away from the surface in the intermediate temperature regime. The decomposition of these clusters in solution at higher temperatures leads to smoother surfaces, but the overall growth rate is smaller. Experimentally, stirring the solution during growth may hinder the reattachment of clusters in the intermediate temperature regime and further optimize growth conditions. In the future, we plan to extend our approach to other types of MOFs containing more than two types of building units.

## Data Availability

Not applicable.

## Supplementary Materials

The following are available online at https://www.mdpi.com/article/10.3390/nano11071631/s1, Figure S1: (a) Distribution of patchy sites D1, D2 and D3 around the mass center R1; (b) distribution of patchy sites O1 and O2 around the mass center R2. All masses of corresponding building units are concentrated to their rigid center R1 and R2. Attractive patchy pairs D1-O1 and D2-O2 attract each other, transparent pair O2-O2 does not interact, while the remaining repulsive pairs repulse each other to prevent overlapping, Figure S2: A schematic illustration of the possible connections of BTCs to the top and bottom of SBU, in the absence of patchy site D3, Figure S3. RDF results at different reactant concentrations. (a) Comparison of RDF distribution of R1-R1; (b) comparison of RDF distribution of R2-R2; (c) comparison of RDF distribution of R1-R2; (d) enlarged RDF distribution of R1-R1; (e) enlarged RDF distribution of R2-R2; (f) enlarged RDF distribution of R1-R2; the peak intensity and peak width of the lower temperature cases have been weakened and broadened to varying degrees comparing to the structure obtained at 25 °C, Figure S4: RDF results at different temperatures. (a) Comparison of RDF distribution of R1-R1; (b) comparison of RDF distribution of R2-R2; (c) comparison of RDF distribution of R1-R2; (d) enlarged RDF distribution of R1-R1; (e) enlarged RDF distribution of R2-R2; (f) enlarged RDF.
