# Supplementary material for: Modeling the Layer-by-Layer Growth of HKUST-1 Metal-Organic Framework Thin Films
Source: Nanomaterials (Basel). 2021 Jun 22;11(7):1631. doi: 10.3390/nano11071631 (PMC8304807; doi:10.3390/nano11071631)
Supplement: Supplementary file 1 [file nanomaterials-11-01631-s001.zip › nanomaterials-1252484-supplementary.pdf]

Supplementary Materials

Modeling the Layer-by-Layer Growth of HKUST-1 Metal-Organic Framework Thin Films

Qiang Zhang [1], Yohanes Pramudya [2], Wolfgang Wenzel [2] and Christof Wöll [2,*]

[1] Institute of Functional Interfaces, Karlsruhe Institute of Technology, 76344 Eggenstein-Leopoldshafen, Germany; qiang.zhang@kit.edu
[2] Institute of Nanotechnology, Karlsruhe Institute of Technology, 76344 Eggenstein-Leopoldshafen, Germany; yohanes.pramudya@kit.edu (Y.P.); wolfgang.wenzel@kit.edu (W.W.)
* Correspondence: christof.woell@kit.edu

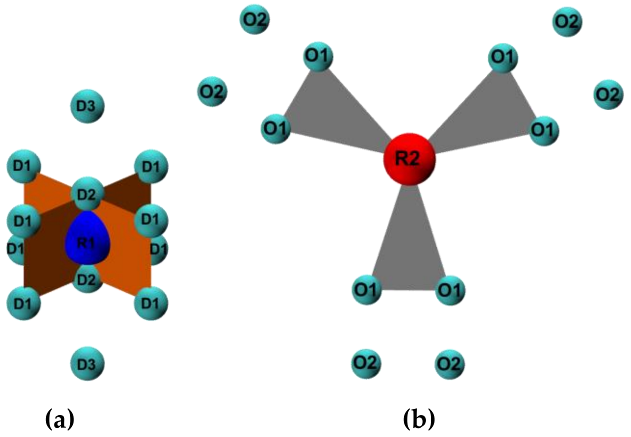

Figure S1. (a) Distribution of patchy sites D1, D2 and D3 around the mass center R1; (b) Distribution of patchy sites O1 and O2 around the mass center R2. All mass of corresponding building units are concentrated to their rigid center R1 and R2. Attractive patchy pairs D1-O1 and D2-O2 attract each other, transparent pair O2-O2 does not interact, while the remaining repulsive pairs repulse each other to prevent overlapping.

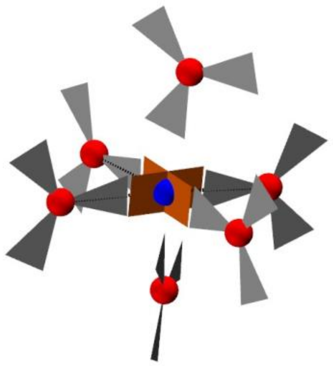

Figure S2. A schematic illustration of the possible connections of BTCs to the top and bottom of SBU, in the absence of patchy site D3.

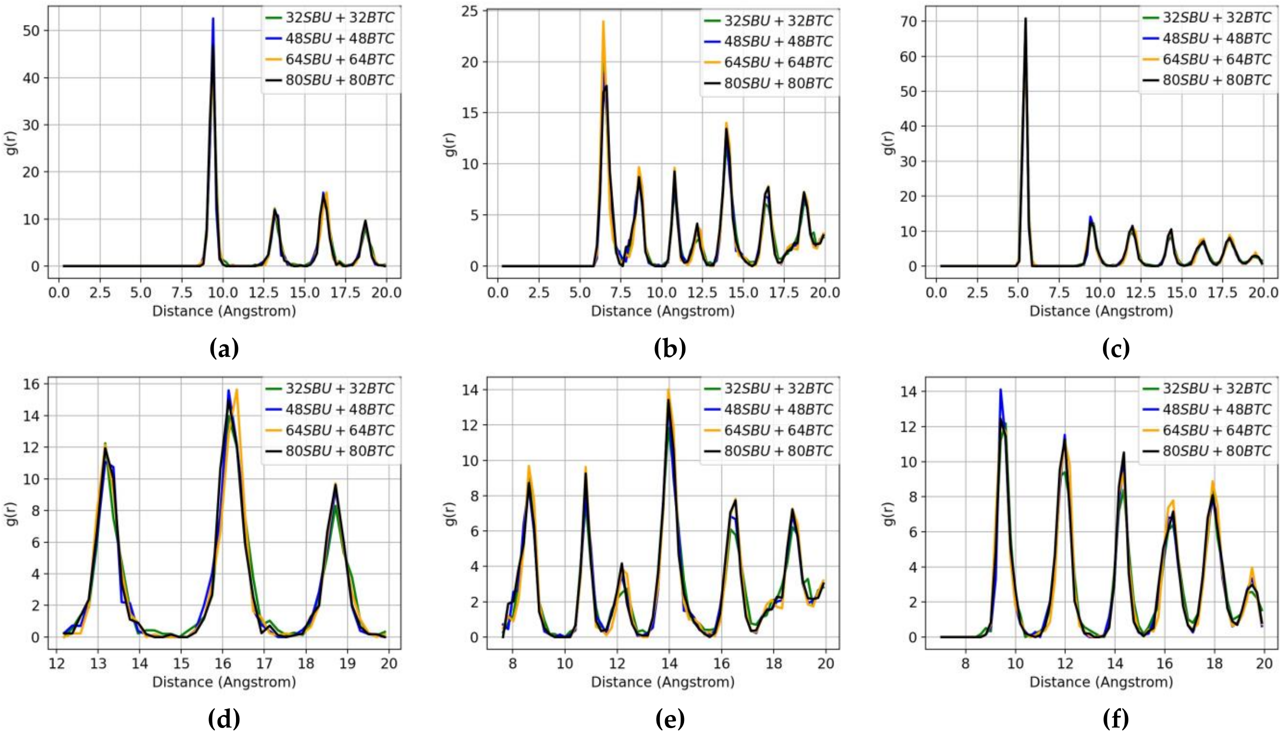

Figure S3. RDF results at different reactant concentrations. (a) Comparison of RDF distribution of R1-R1; (b) Comparison of RDF distribution of R2-R2; (c) Comparison of RDF distribution of R1-R2; (d) Enlarged RDF distribution of R1-R1; (e) Enlarged RDF distribution of R2-R2; (f) Enlarged RDF distribution of R1-R2; The peak intensity and peak width of the lower temperature cases have been weakened and broadened to varying degrees comparing to the structure obtained at 25 °C.

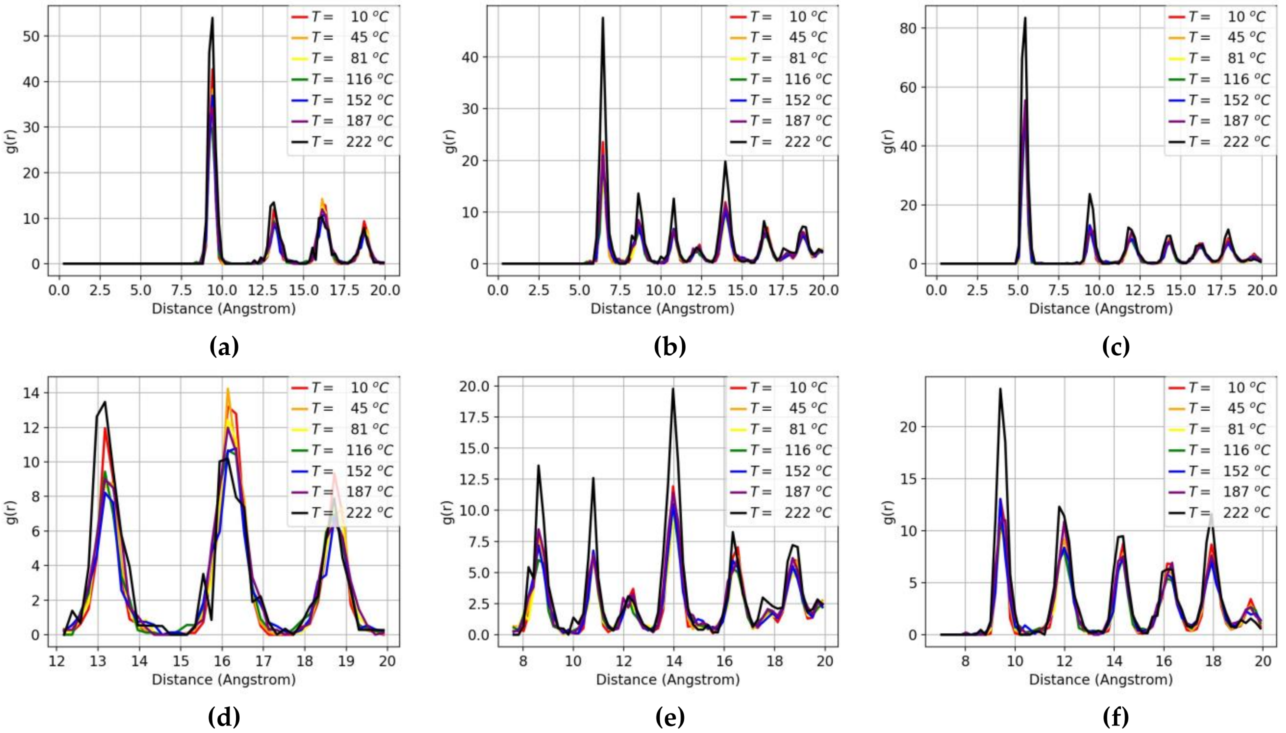

Figure S4. RDF results at different temperatures. (a) Comparison of RDF distribution of R1-R1; (b) Comparison of RDF distribution of R2-R2; (c) Comparison of RDF distribution of R1-R2; (d) Enlarged RDF distribution of R1-R1; (e) Enlarged RDF distribution of R2-R2; (f) Enlarged RDF.
